# Preparation and Distribution of Cannabis and Cannabis-Derived Dosage Formulations for Investigational and Therapeutic Use in the United States

**DOI:** 10.3389/fphar.2016.00285

**Published:** 2016-08-31

**Authors:** Brian F. Thomas, Gerald T. Pollard

**Affiliations:** ^1^Discovery Sciences, Research Triangle Institute, Research Triangle ParkDurham, NC, USA; ^2^Howard Associates, LLC, Research Triangle ParkDurham, NC, USA

**Keywords:** cannabis, cannabinoids, marijuana, Δ^9^-tetrahydrocannabinol (Δ^9^-THC), cannabidiol, DEA controlled substances, therapeutics, quality control

## Abstract

Cannabis is classified as a schedule I controlled substance by the US Drug Enforcement Agency, meaning that it has no medicinal value. Production is legally restricted to a single supplier at the University of Mississippi, and distribution to researchers is tightly controlled. However, a majority of the population is estimated to believe that cannabis has legitimate medical or recreational value, numerous states have legalized or decriminalized possession to some degree, and the federal government does not strictly enforce its law and is considering rescheduling. The explosive increase in open sale and use of herbal cannabis and its products has occurred with widely variable and in many cases grossly inadequate quality control at all levels—growing, processing, storage, distribution, and use. This paper discusses elements of the analytical and regulatory system that need to be put in place to ensure standardization for the researcher and to reduce the hazards of contamination, overdosing, and underdosing for the end-user.

*Cannabis sativa* L. is a widespread species in nature, and its use in medicine, food, textiles, recreation, and religion dates back thousands of years. The pharmacology of its major constituent phytocannabinoids, notably Δ^9^-tetrahydrocannabinol (Δ^9^-THC) and cannabidiol (CBD), has been extensively investigated. Other pharmacologically active phytocannabinoids as well as terpenes, flavonoids, and other constituents that may contribute to therapeutic efficacy or adverse side effects, including abuse liability, have not been so well studied.

The United States (US) cannabis market is expected to grow to $7.1 billion in 2016, a 26% increase over 2015 (ArcView Market Research New Frontier, 2016). While the diversity of cannabis dosage formulations and medical claims continues to increase dramatically, there remains considerable uncertainty about phenotypes, chemotypes, naming conventions, dosage formulation labeling accuracy, consistency, quality control, and the impact of chemical diversity and variation in chemical composition on pharmacological or adverse effect end-points. For instance, the Food and Drug Administration (FDA) issued several warning letters in 2015 and again in 2016 about inappropriate and illegal medical claims for CBD formulations manufactured or distributed by US companies. They cited instances where the manufacturer or distributor claimed “therapeutically active” concentrations of CBD but, upon analytical testing in FDA laboratories, no active ingredient was found. A variety of formulations purchased in state dispensaries failed to meet basic label accuracy standards for pharmaceuticals. In one study, more than 50% of products evaluated had significantly less cannabinoid content than indicated on the label, with some containing negligible amounts. Other products had significantly more THC than labeled, placing patients at risk of adverse effects, including drug interactions, and off-target effects (Vandrey et al., 2015). In addition to inaccurate concentrations, there is concern about the presence of other matter not listed on the label, such as pesticides, fertilizers, fungicides, rodenticides, molds, microbes, heavy metals, and other chemical adulterants that may be health and environmental hazards.

Consistency in the chemical constituents of feedstock material and finished dosage formulations is a basic requirement in the pharmaceutical industry for quality, safety, and efficacy of botanically derived drugs. Herbal cannabis as a manufacturing feedstock presents a particular challenge for consistency because it is chemically complex and variable as a result of its allogamous nature. The chemical composition of cannabis biomass is further affected by environment, development (age), sex, and post-harvest processing and storage conditions. The chemical consistency of cannabis feedstock for pharmaceuticals is best ensured through good laboratory and agricultural practices, including selection of appropriate female clones based on careful analytical characterization of chemical composition, botanical conservation, mass multiplication using advanced biotechnological methods, and consistent harvesting and post-processing techniques (Thomas and ElSohly, 2015). Indeed, a variety of cannabis strains have been selected for certain physical (phenotype) or chemical (chemotype) attributes over time, and the processing and development of dosage formulations has expanded dramatically, resulting in a plethora of product types available to the recreational user, nutritional consumer, and patient. For safe and effective use, this expanding range of strains and formulations should be standardized and subjected to rigorous analysis of active ingredients, impurities, and degradants at all stages of processing and manufacturing, so that finished drug products are characterized for homogeneity, stability, and drug delivery over time and conditions of anticipated storage and use.

## Production of botanical drug products

Many nations and US states have recently revised their regulatory control of the production, distribution, and use of herbal cannabis and its dosage formulations. A majority of the US states have decriminalized some possession offenses, or legalized recreational or medicinal use, in direct violation of existing federal regulations. According to current federal statutes and treaties, the bulk manufacture of cannabis and its formulations in the US is controlled by the Drug Enforcement Administration (DEA) in accordance with the Single Convention on Narcotic Drugs and the Controlled Substances Act (CSA), in which they are schedule I, the most restrictive classification. The CSA requires the DEA to limit the number of bulk manufacturers to the minimum required for an adequate and uninterrupted supply of cannabis-derived substances under adequately competitive conditions to support legitimate medical, scientific, research, and industrial purposes [21 U.S.C. §823(a)(l)]. Under the Single Convention, the DEA must also ensure that manufacturers are appropriately registered and adhere to the system of controls required by the treaty, which includes maintaining a monopoly on the distribution of material for research. The US has satisfied its treaty obligation through an arrangement where the National Institute on Drug Abuse (NIDA) oversees the domestic production and distribution of cannabis by its contractors, the University of Mississippi and Research Triangle Institute (RTI), respectively^1^. The amount manufactured by the University of Mississippi is controlled through yearly aggregate production quotas issued by the DEA. The DEA works with NIDA to set the annual quota to meet research needs of the US, and may include production of a particular strain or specific extracts or purified natural products. DEA regulations also allow a bulk manufacturer to maintain an inventory equal to 50% of its average estimated net disposal for the current calendar year; these quotas can be adjusted by the DEA.

The DEA can increase the number of entities registered as bulk manufacturers as long as the requirements of the CSA and Single Convention are met. These requirements include a stipulation that the number of bulk manufacturers be kept to the minimum required to supply authorized recipients, and thus will not be increased until it is determined by the DEA and NIDA that adequate amounts or varieties of plant material cannot be provided by the University of Mississippi. Any additional registered growers would have to be acting under the direct control of the US Government with respect to production and distribution. Interestingly, the DEA has received only one application by a person seeking to become a registered bulk manufacturer to supply researchers, in addition to the University of Mississippi, since the enactment of the CSA in 1970. The decision by the DEA to deny the application was upheld by the US Court of Appeals for the First Circuit in Craker v. DEA, 714 F.3d 17 (1st Cir.2013). However, in response to changing public opinion on recreational and medicinal use and the resultant increased demand for research, the government has acted to expand legal production at the University of Mississippi. In 2014, the annual amount was increased over 10-fold at the request of NIDA, and the crop included new strains differing in their ratios of primary phytocannabinoids.

No pesticides or herbicides are used on the crop at the University of Mississippi, but assurances cannot be made for the products being sold in US dispensaries. The market value is so great that some growers use a chemical arsenal to feed and protect the crop from microbial and animal infestation and disease to realize the largest yield possible. Because the US government still considers cannabis manufacture illegal outside of that contracted by NIDA at the University of Mississippi, the Environmental Protection Agency (EPA) cannot research or register pesticides, fungicides, or herbicides for this use. However, section 24(c) of the Federal Insecticide, Fungicide, and Rodenticide Act (FIFRA) permits states with unique or unusual pest management challenges to seek a special local need (SLN) registration of a pesticide when a national registration may not be necessary or appropriate. States that issue section 24(c) registrations must submit them to EPA for review. The information the EPA expects states to consider in assessing human safety and environmental risks from application of the pesticide are described at 40 C.F.R. 162. If the EPA does not disapprove the state's SLN registration, distribution, and use can proceed in the registering state with the label specifying the particular use or crop. However, as of January 2016, the EPA had not received any SLN registrations for cannabis. The absence of approved standards for use, limits on chemicals types and residues, and validated testing methods for cannabis products may result in consumer exposure to hazardous agents or higher residue levels than would occur if regulatory guidance and specifications existed (Stone, 2014). Administration by smoking and inhalation may also present greater risk of exposure with certain chemicals compared to ingestion. Moreover, extraction and concentration methods used for the manufacture of some dosage formulations (tinctures, butane hash oil, “dabs”) can result in final products containing high levels of residual solvents and pesticides, which has been verified through residue monitoring (Raber et al., 2015), enforcement activities, and legislative testimony (Stone, 2014). The use of fertilizers, pesticides, herbicides, and fungicides also poses environmental risk (Scott Nolen, 2014; Carah et al., 2015). The consumer should recognize the potential risks from cannabis obtained through dispensaries.

## Manufacture of dosage formulations

Rigorous quality control of feedstock and batch production processes using good laboratory and manufacturing practices and validated analytical methods is required for consistency in dosage formulation and suitability of finished product for its intended purposes, particularly when therapeutic efficacy, and safety are being assessed. Sampling and analytical characterization methods before, during and after production are specific to the nature of the product and its processes.

When bulk material is going to be consumed simply through combustion or vaporization, it is still processed to some degree. This is done because phytocannabinoid content varies widely across the entire plant, leading to greater potential for variance in dose, and effect. Concentration and homogeneity can be dramatically increased when cloned, non-fertilized female plants are grown indoors under controlled conditions, harvested at the optimal time of flowering and senescence, and carefully manicured to isolate the trichrome-rich flowers (inflorescence). The inflorescence must be dried, processed, and packaged to deter fungal and microbial growth, prevent contamination and pest infestation, and protect the material from light and environmental exposure. The drying process to between 5 and 10% can be expedited by maintaining low humidity and moderate temperature (20–40°C) to reduce the risk of mold while maintaining the terpene constiuents of the inflorescence. Over extended aging time, decarboxylation converts the phytocannabinoid acids, and terpenes isomerize to create more complex polyterpenes with unique tastes and aromas (Upton et al., 2013). The cannabis harvested at the University of Mississippi for research or medical use is immediately processed, dried, and packaged in FDA-approved polyethylene bags, and stored in the dark at −20°C until ready for dosage manufacture or distribution in bulk. Researchers and patients may rehydrate cigarettes that have been produced as NIDA dosage formulations to increase their moisture content to between 10 and 12% before consumption.

Dosage formulations, including cannabis cigarettes and their placebos, cannabis tinctures, and purified cannabinoids are available to NIH researchers. The production of large quantities of cigarettes requires the use of a modified tobacco cigarette manufacturing machine to produce standardized, non-filter cannabis or placebo cigarettes of uniform weight and desired specifications. To facilitate manufacture, the bulk plant material needs to be processed to acceptable particle size and consistency. Placebo material can be prepared by repeated solvent (typically ethanol) extraction and used in bulk or in cigarette manufacture. Alternatively, cigarettes can be manufactured from processed cannabis in smaller scale using table-top cigarette maker machines. Several million cigarettes in various phytocannabinoid concentrations and placebos have been manufactured in this way and are available for use through NIDA as medicinal dosage formulations or for research. Extracts, tinctures, and purified phytocannabinoid preparations have been produced for use as requested by researchers and authorized by NIDA. Several types of extracts and purified phytocannabinoids have been studied. During the batch production process, through the use of moderate heat over an extended time, the acid forms can be isolated intact or intentionally decarboxylated to as great an extent as possible while avoiding further thermal decomposition to unwanted degradants. Sterile processing and filtration can be used to manufacture formulations for intravenous administration, and several sterile formulations have been developed and distributed through the NIDA Drug Supply Program (DSP). Butane hash oil and supercritical fluid extracts are concentrated forms available at cannabis dispensaries. The illicit manufacture of butane hash oil has been associated with fires and severe injuries. While this formulation has not been manufactured for use in the NIDA DSP, RTI is developing supercritical fluid capabilities that can extract and purify cannabis (and tobacco) constituents at the preparative scale. Other products developed or investigated at RTI for NIDA are oral dosage formulations in sesame oil, ethanol, and Tween-80 for human use (Perez-Reyes et al., 1973); and edible formulations have been produced with the help of some practical knowledge (researchers might recall Dr. Mario-Perez Reyes's unpublished studies with cookies). Whatever the form, process control samples taken before, during, and after production are characterized analytically to monitor physical (weight) and chemical (phytocannabinoid concentration) parameters, and to determine batch uniformity and other characteristics required for the datasheets and certificates of analysis that are distributed with the materials. All processes are reviewed by quality control systems, and human use materials are subject to further review and release by quality assurance systems as described in detail below.

## Analytical characterization and stability testing

Upon receipt of cannabis plant material for manufacture, the biomass is quarantined in secure storage facilities approved for schedule I use by the DEA, and conditionally released for analytical characterization to confirm identity and potency (strength). The raw material is sampled for homogeneity and characterized for potency through quantitative measurement of its most active or abundant phytocannabinoids, many of which may be present in their biosynthetic acid or decarboxylated forms, such as Δ^9^-THC, CBD, tetrahydrocannabivarin (THCV), cannabinol (CBN), cannabigerol (CBG), and cannabichromene (CBC). Limit tests must be performed for pesticides and hazardous chemicals used in production or suspected to be present, and the microbial burden must be adequately assessed and controlled. Moisture content is an important parameter affecting storage stability and processing, and other chemical content may be measured as warranted. For example, terpenes such as α-pinene and limonene provide characteristic organoleptic (taste and aroma) profiles in cannabis strains, but may also contribute to pharmacological profiles through their actions at transient receptor potential channels or other biochemical enzymes or substrates, including cannabinoid receptors (β-caryophyllene is an example); hence the current interest in terpenes.

After conditional release for manufacture, batch production proceeds in an appropriate facility and is recorded in a batch production record using appropriate process controls and documented equipment calibration and performance verification procedures. In some instances, analytical information must be obtained before completion of processing. Time-zero samples are taken from the final product by documented procedures, analyzed for batch uniformity, and stored in qualified stability chambers or storage facilities where temperature and humidity are monitored and controlled over varying storage times. Critical performance specifications for dosage formulations are tested and documented on datasheets supplied to researchers, such as weight, potency, and unique parameters for particular formulations. Cigarette smoke yields can be assessed by validated smoking machines. All analytical data and other documentation are reviewed by an independent quality control chemist and by quality assurance people prior to release, with each product accompanied by its informational datasheets. All materials within the NIDA DSP are also subject to intermittent quality control analysis over time to ensure suitability for use. In some instances, materials, or formulations may need to be purified periodically. Distribution to researchers can occur within a few days of shipment authorization, though some instances may require 2–3 weeks to meet a researcher's individual needs.

## Distribution of cannabinoid dosage formulations

Researchers who wish to do studies with cannabis must have a special DEA registration under the CSA [21 U.S.C. § 823(f) and 21 CFR 1301.18 and 1301.32]. Application is submitted on DEA Form-225 for the appropriate schedule I drug code, along with a 1-year registration fee, a research protocol showing the amount of drug needed, and a copy of the researcher's curriculum vitae. Upon receipt of the application, a DEA investigator conducts a site visit to verify that security procedures and diversion controls are adequate for storage and use. The DEA also sends the research protocol to the FDA for review and approval. The researcher might also need to register with state and local agencies and comply with their regulations for schedule I.

US researchers with NIH grant funding submit a request to NIDA's DSP along with a copy of their DEA registration, a completed DEA Form-222, and other required documentation about funding and intended use. Investigators who desire access to human use material from the DSP must also have documentation of local institutional review board approval and an active Investigational New Drug Application (IND) for the conduct of clinical studies that has been evaluated and found safe by the FDA. NIDA has compiled a Drug Master File that provides the FDA with information on seed selection, growth, harvesting, manufacturing, analytical characterization, and stability testing for each batch of its cannabis and formulations, which can be referenced in the IND to facilitate use in human subjects. If the material is to be used for non-clinical human research, the request is simply forwarded to NIDA's Office of the Director for review and recommendation. Researchers without an NIH grant are subject to additional scientific review. Foreign applicants must provide documentation that controlled substances, research chemicals, or cannabis cigarettes being requested are permitted for import into their countries.

When NIDA approves and authorizes the distribution of a schedule I controlled substance to a researcher or patient, materials are released, packaged, and distributed for use in preclinical and clinical studies or for medicinal use under the Compassionate Use Act. A barcode-based electronic inventory management system tracks every batch, providing independent, automated verification of all compound supplies, allocations, and distributions. Release of materials classified as “Animal Use” or “Other” is based on examination and verification that they are exactly as described in the authorizing documents; this is checked by a second person, noted on the shipment document, and checked again by the person who packages the shipment. Materials classified as “Human Use” require release by quality assurance staff, who are responsible for oversight of all processes involved with human-use materials to ensure good practice quality guidelines and regulations (GxP) compliance. Each batch has a certificate of analysis or a data sheet that is provided to the researcher or end user containing information on the particular batch along with recommendations for its proper handling, storage, and use. Packaging must comply with federal code and is customized to minimize extremes of temperature and provide maximum protection against breakage, contamination, and loss. When refrigeration is required, material may be shipped on cold packs at −20 or on dry ice at −70°C.

From the time a researcher submits a request to NIDA, the entire review, authorization, shipment preparation, release, and distribution can typically be done within 2–4 weeks. For materials to be exported, an export permit must be obtained from the DEA and an import permit from the destination country for each shipment, along with a statement that the drugs will not be re-exported. If all of these documents are found by the DEA to be correct, an export permit will typically be issued in 2–3 weeks.

## The evolving role of the DEA, NIDA, and FDA in cannabinoid research

Medicinal and recreational users and research investigators often desire access to higher potency materials and more varieties of cannabis and cannabinoid dosage formulations than are currently available through NIDA (Russo et al., 2002; Stith and Vigil, 2016), and efforts to address these unmet needs are ongoing (Reardon, 2015). However, the limitations in the NIDA DSP inventory are compounded by the rapid proliferation of cannabis varieties, cannabis-derived formulations (e.g., lipstick, creams, sunscreen, edibles, and drinks), and routes of administration (dermal, vaporization, and inhalation). Many of these products that are commercially available on the illicit market or in state dispensaries are not sufficiently characterized to enable distribution to researchers through the NIDA drug supply program. Thus, NIDA cannabis and cannabis-derived products may not be representative of cannabis in commerce, whether intended for recreational or therapeutic use (see Bloor et al., 2008; Shen, 2014; ElSohly et al., 2016, for example). Moreover, while polls suggest that most people believe cannabis has therapeutic utility and should not be schedule I, the DEA has rejected rescheduling petitions several times in the past; most recently in August of 2016. Rescheduling would enable doctors to prescribe cannabis and cannabis-derived botanical drugs as therapeutics. It would also facilitate research by professionals to investigate the basic science of cannabis, and to use this information to better understand neurophysiological function, develop new medicines for people and animals, and find improved ways to deal with abuse. Rescheduling or de-scheduling might also decrease the abuse of the novel psychoactive synthetic cannabinoids that have been sold as legal “incense,” and that pose significant health risks and challenges for detection of use. Thus, the current status of cannabis use in the US continues to present unique policy, regulatory, criminal justice, financial, and research concerns that can only be legally addressed with the assistance and support of the DEA, NIDA, and the FDA. Working together, these agencies, along with researchers, physicians, patients, and industry stakeholders, can help ensure the proper manufacture, labeling, and distribution of safe and consistent products with known chemical content and well-characterized performance. More information on access to cannabis can be obtained from NIDA (https://www.drugabuse.gov/researchers/research-resources/nida-drug-supply-program), the DEA (https://www.dea.gov/divisions/hq/2016/hq081116.shtml), and the FDA (http://www.fda.gov/NewsEvents/PublicHealthFocus/ucm421168.htm).

## Author contributions

BT prepared the draft outline and contributed to the preparation of the manuscript. GP also contributed to the preparation and editing of the manuscript.

## Funding

This work was supported by the National Institutes of Health National Institute on Drug Abuse (Contract HHSN271201500007C and Grant R01DA-040460).

### Conflict of interest statement

The authors declare that the research was conducted in the absence of any commercial or financial relationships that could be construed as a potential conflict of interest.

## Footnotes

^1^The lead author of this perspective (BFT) serves as the Principal Investigator on the NIDA Drug Supply Program contract entitled “Preparation and Distribution of Research Drug Products,” which provides for the manufacture and distribution of cannabis dosage formulations, and as a subcontractor to the University of Mississippi on their NIDA contract entitled “Production, Analysis and Distribution of Cannabis, Marijuana Cigarettes, and Related Materials.” The opinions and conclusions expressed in this publication are those of the authors and do not necessarily reflect those of NIDA or the DEA.
